# Does Age Matter? A Comparison of Personality Profiles in Adult and Older Patients with Cancer Using the Structural Analysis of Social Behavior (SASB)

**DOI:** 10.3390/healthcare14162452

**Published:** 2026-08-08

**Authors:** Roberta Spatuzzi, Maria Velia Giulietti, Paolo Fabbietti, Anna Vespa

**Affiliations:** 1 Department of Mental Health, ASP Basilicata, 85100 Potenza, Italy; 2 Department of Neurology, INRCA-IRCCS National Institute of Health and Science on Aging, 60124 Ancona, Italy; giulietti.mariavelia@libero.it; 3 Biostatistical Center, INRCA-IRCCS National Institute of Science and Health on Aging, 60124 Ancona, Italy; p.fabbietti@inrca.it; 4 Psychotherapist and Senior Researcher, INRCA-IRCCS National Institute of Health and Science on Aging, 60124 Ancona, Italy; a.vespa@inrca.it

**Keywords:** personality, intrapsychic behaviors, SASB, aging, cancer

## Abstract

**Highlights:**

**What are the main findings?**

- At the unadjusted level, significant age-group differences emerged only in SASB Cluster 1, with higher scores observed among older cancer patients.
- Older and adult patients differed significantly in marital status and education, highlighting distinct cohort-specific backgrounds.

**What are the implications of the main findings?**

- Personality functioning in cancer patients should be interpreted within a socio-demographic and life-course framework rather than based on chronological age alone.
- Psycho-oncological assessment and interventions should adopt a personalized, lifespan-oriented approach that integrates personality functioning with patients’ socio-demographic characteristics.

**Abstract:**

**Background/Objectives:** This study aimed to examine personality profiles at the intrapsychic level (“Oneself”) in adult and older patients with cancer and to compare potential differences between these age groups. **Methods:** In this observational, cross-sectional comparative study, 273 patients with cancer (all disease stages) were categorized into two age groups: adults (40–64 years, *n* = 141) and older adults (≥65 years, n = 132). Personality was assessed using the Structural Analysis of Social Behavior (SASB) Form-A. Descriptive and inferential bivariate statistical analyses, including Chi-square tests and independent-samples *t*-tests, were performed to evaluate differences between the two age groups. To further examine the association between age group and SASB Cluster 1, a multivariable exploratory post hoc factorial General Linear Model (factorial GLM) was conducted. **Results:** Comparisons between adults and older adults revealed significant differences in socio-demographic backgrounds, with older patients being more frequently widowed and having lower educational attainment (both *p* < 0.001). Regarding personality dimensions, a significant difference was observed exclusively in SASB Cluster 1 (Autonomy-Assertive and Separating), with higher scores among older participants (4.4 vs. 3.9; *p* = 0.002). However, in the adjusted factorial GLM, the main effect of age group was no longer statistically significant (*p* = 0.054), and no significant two-way interactions emerged, including the age-group-by-education interaction (*p* = 0.222). This indicates that the initial unadjusted age-group association was attenuated after accounting for selected socio-demographic variables. No significant differences were found between the two groups in the remaining SASB Clusters. **Conclusions:** These findings caution against overestimating the role of chronological age as an isolated variable in psycho-oncological research. Personality differences in Cluster 1 co-occur with distinct cohort-specific and socio-demographic life-course profiles. Understanding how personality relates to shifting socio-demographic circumstances across the lifespan may support the development of more tailored, context-sensitive psycho-oncological interventions.

## 1. Introduction

Population aging is one of the most significant demographic trends worldwide [1]. Improvements in healthcare and living conditions have led to a substantial increase in life expectancy, resulting in a growing proportion of older adults in the population. Because cancer incidence rises markedly with age, cancer occurrence increases from mid-adulthood onwards, with most cases diagnosed in middle-aged and older adults and more than half occurring in individuals aged 65 years and older [2]. Consequently, cancer is widely regarded as a disease strongly associated with aging [3].

Although research addressing psychological differences across age groups in oncology remains relatively limited, available evidence suggests that adults with cancer tend to report higher levels of cancer-related distress [4], poorer perceived quality of life [5], and greater post-traumatic stress symptomatology compared to older patients [6]. This heightened vulnerability has been linked to the disruption of key developmental tasks that typically occur in early and middle adulthood, such as career establishment, family formation, and financial stability [7,8].

In contrast, older adults with cancer may present a different pattern of psychological adjustment. Although, on average, they tend to report lower levels of emotional distress compared to adult patients, they are often exposed to cumulative age-related stressors, including widowhood, social isolation, multimorbidity, and declining physical functioning [9,10]. The co-occurrence of these conditions with a cancer diagnosis may create a “double whammy” effect, resulting from the overlap between age-related vulnerabilities and disease-related burden, thereby increasing clinical complexity and psychosocial care needs [9].

At the same time, aging may also encompass adaptive resources that can facilitate coping with illness. Greater life experience, repeated exposure to adverse events, and a more consolidated capacity to derive meaning from difficulties may support emotional regulation in the context of serious disease [11]. In addition, older patients are often less exposed to competing social and occupational demands compared to younger individuals, who are more frequently engaged in simultaneous work, family, and financial responsibilities [12]. These contextual factors may contribute to more stable and less reactive patterns of emotional adjustment in later life, potentially buffering against psychological distress despite the presence of significant health-related challenges.

Consistent with this perspective, differences in coping strategies have been observed across the adult lifespan. Adult patients are more likely to rely on active problem-focused coping strategies, whereas older patients tend to show greater acceptance of illness-related limitations and a more emotionally regulated response style [13]. Together, these findings suggest that psychological adjustment to cancer may vary across age groups, although such differences are likely influenced by developmental, social, and contextual factors.

Among the psychological factors that may contribute to these age-related differences, personality has received growing attention in psycho-oncology research. Personality represents a relatively stable configuration of psychological characteristics that shape how individuals perceive themselves, relate to others, regulate emotions, and respond to environmental demands [14,15]. Within the context of cancer, personality traits may influence how patients interpret their diagnosis, engage with treatment, and adapt to the physical and psychological consequences of the disease [16]. As such, personality may represent a key factor in explaining variability in psychological adjustment and quality of life among cancer patients [17]. However, despite extensive theoretical and empirical work, the precise nature of the relationship between personality and cancer remains only partially understood. In particular, less is known about how personality characteristics may interact with age-related differences in psychological functioning within oncology populations.

To address these issues, the present study adopts the Structural Analysis of Social Behavior (SASB) [18,19,20], a circumplex model that provides an integrative assessment of both intrapsychic and interpersonal dimensions of personality. The SASB framework organizes behavior along the dimensions of affiliation and interdependence, and identifies distinct clusters of psychological functioning that capture both adaptive and maladaptive patterns. This model has been widely applied in personality assessment, psychopathology research, and psychotherapy outcome evaluation, offering a comprehensive framework for conceptualizing personality as a dynamic and interconnected system [20]. Compared with traditional trait-based personality models, the SASB model provides a specific focus on interpersonal functioning and self-other representations, allowing the assessment of how individuals relate to themselves and others across different relational contexts. This perspective is particularly relevant in oncology, where illness-related experiences may influence both self-perception and interpersonal relationships.

In light of the limited evidence regarding age-related differences in personality functioning among oncology patients, the present study aims to examine SASB intrapsychic patterns (“Oneself”) across different age groups in a sample of cancer patients. A better understanding of these differences may contribute to identifying age-specific psychological needs and to the development of more tailored psychotherapeutic and psychosocial interventions within multidisciplinary cancer care.

## 2. Materials and Methods

### 2.1. Study Design, Setting, and Participants

The present research used an observational, cross-sectional, and comparative study design aimed at investigating age-related profiles in intrapsychic personality functioning among oncological patients.

A total of 445 patients with cancer were initially approached in the clinic by physicians and invited to participate in this comparative study. The recruitment was conducted by oncologists in the Oncology Departments of INRCA-IRCCS (National Institute of Health and Science on Aging) and in the Endocrinology Department of the United Hospitals of the Polytechnic University of Marche in Ancona, Italy. Patients had been affected by cancer for 1 to 9 years.

Inclusion criteria included: age between 40 and 80 years; ability to understand the Italian language; histologically confirmed cancer diagnosis (biopsy); and provision of written informed consent to participate in the study. Patients were excluded if they refused to participate, were unable to provide informed consent, had more than one type of cancer, had a psychiatric history, or had a previous history of malignancy, with the exception of non-melanoma skin cancers.

Of the 445 patients approached, 350 agreed to participate and signed the informed consent form after a detailed explanation of the study protocol provided by the physician at the oncology clinic. Splitting the sample by complete data availability and inclusion criteria, 77 patients were excluded because they did not answer all questionnaire items and/or did not strictly meet the inclusion criteria. Therefore, the final study sample consisted of 273 patients. For the purpose of age-group comparisons, participants were categorized into two subgroups: adult patients aged < 65 years (*n* = 141) and older patients aged ≥ 65 years (*n* = 132). This threshold was defined a priori, as it is widely adopted in epidemiological and clinical oncology research to distinguish older adult cancer patients from younger adult populations and is commonly used in age-specific oncology frameworks [21].

Participants were interviewed by two psychotherapists working in the hospital, each with one year of training in the administration and interpretation of the SASB model, following histological diagnosis. Patients completed all questionnaires, including socio-demographic (age, marital status, and educational level) and clinical variables, during the interview. The study was approved by the Ethics Committees of the INRCA-IRCCS National Institute of Science and Health for Aging and the Polytechnic University of Marche in Ancona, Italy (IRB approval number: CdB: SC/10/271/bis).

### 2.2. Sample Size

An a priori power analysis was conducted using G*Power (version 3.1.9.7) to determine the minimum sample size required for the primary statistical analyses. To detect a medium effect size (Cohen’s d = 0.50) in the comparison of SASB cluster scores between the two age groups using a two-tailed independent-samples *t*-test, with alpha = 0.05 and a statistical power of 0.80, a minimum total sample size of 128 participants (64 per group) was required. Additionally, for the socio-demographic and clinical contingency tables, a sample size of 122 participants was sufficient to detect a medium effect size (Cohen’s w = 0.30) in a Chi-square test with up to 3 degrees of freedom. The final study sample (*n* = 273), comprising 141 adult patients and 132 older patients, strictly met and exceeded these requirements, ensuring adequate statistical power for all planned bivariate comparisons.

### 2.3. Ethical Statement

The study was conducted in accordance with the Declaration of Helsinki and approved by the Ethics Committees of INRCA-IRCCS and Marche Polytechnic University, Ancona, Italy (protocol code: CdB: SC/10/271/bis). Prior to data collection, written informed consent was acquired from all participants.

### 2.4. Instruments

#### 2.4.1. Demographic and Clinical Data

Clinical and sociodemographic variables were obtained through a *social schedule* designed by the researchers. The collected data included information on age, marital status (single, married, widowed, divorced), educational level (illiterate, primary school, middle school, high school, university), and cancer stage (I–IV).

#### 2.4.2. The SASB Intrex Questionnaire

The SASB Intrex Questionnaire [18,19,20,22,23] describes personality structure at both intrapsychic and interpersonal levels (Supplementary Materials). This instrument was selected for its brevity and because it demonstrates adequate reliability and validity in assessing interpersonal and intrapsychic behaviors, and it is validated within frameworks consistent with DSM-IV and DSM-5 criteria.

Participants were required to respond to 36 items assessing their intrapsychic and interpersonal behaviors over the previous year (e.g., “I let myself feel glad about and pleased with myself just as I am”; “I ignore and don’t bother to know my real self”; “I think up ways to hurt and destroy myself. I am my own worst enemy”). Responses were rated on a 10-point Likert scale ranging from 0 (Never) to 10 (All the time). The 36 items of Form A are organized through a specific scoring procedure into eight Clusters (Cl) representing intrapsychic “Oneself” and interpersonal “Other” behaviors, providing a comprehensive description of intrapsychic functioning from which interpersonal patterns can also be inferred. Each cluster score ranges from 0 to 10 and is calculated as the mean score of the respective items forming that specific cluster, rather than a summed score, with higher values indicating greater endorsement of the corresponding behavioral pattern. The present study focused exclusively on the intrapsychic dimension (“Oneself” introject surface) of personality functioning; interpersonal dimensions (“Other”) were not directly assessed or statistically analyzed in this research. The Italian validated version of the questionnaire was used in the present study [23]. For the current sample (*N* = 273), the internal consistency of the relevant SASB clusters was evaluated using Cronbach’s alpha, which demonstrated good to excellent reliability coefficients across all scales: α = 0.78 for Cluster 1, α = 0.79 for Cluster 2, α = 0.77 for Cluster 3, α = 0.81 for Cluster 4, α = 0.76 for Cluster 5, α = 0.86 for Cluster 6, α = 0.96 for Cluster 7, and α = 0.96 for Cluster 8.

The 8 clusters of “Oneself” and “Other” are complementary (Supplementary Materials) [18,19].

The 8 clusters of “Oneself”—Intrapsychic experience—are the following:

- SASB-(Cl)1 = Autonomy-Assertive and separating.
- SASB-(Cl)2 = Autonomy and love-Self-accepting and exploring.
- SASB-(Cl)3 = Love -Self-supporting and appreciative.
- SASB-(Cl)4 = Love and control-Self-care and development.
- SASB-(Cl)5 = Control Self-regulating and controlling.
- SASB-(Cl)6 = Control and hate Self-critical and oppressive.
- SASB-(Cl)7 = Hate -Self-refusing and annulling.
- SASB-(Cl)8 = Hate and autonomy-Self-negligent and mentally absent.

### 2.5. Data Analysis

Personality characteristics were assessed using the Structural Analysis of Social Behavior (SASB) Form-A. Continuous variables were summarized using means and standard deviations (SD), whereas categorical variables were expressed as frequencies and percentages. Comparisons between adult patients (<65 years) and older patients (≥65 years) were conducted using descriptive and inferential statistical analyses. Differences in categorical variables, including gender, marital status, educational level, and cancer stage, were examined using the chi-square test or Fisher’s exact test when appropriate. Differences in SASB cluster scores between the two age groups were evaluated using independent-samples *t*-tests.

To further examine the association between age group and SASB Cluster 1, an adjusted multivariable analysis was conducted. Specifically, a factorial General Linear Model (factorial GLM) was fitted, entering SASB Cluster 1 as the dependent variable, and age group (<65 vs. ≥65 years), marital status, and educational level (EDU) as fixed factors, including all main effects and their interactions. Model assumptions, including homogeneity of variances, were verified using Levene’s test. Partial eta-squared was computed as a measure of effect size. Because this target dimension was selected for multivariable modeling based on univariable baseline differences rather than an a priori primary outcome definition, this specific step must be explicitly considered as a data-driven exploratory post hoc analysis. Potential clinical and biological confounders, such as patient sex and tumor stage, were initially excluded from the main factorial model to maintain optimal statistical parsimony. However, to rigorously evaluate the methodological robustness of our findings against potential residual confounding based on clinical and theoretical plausibility, a formal sensitivity analysis was conducted by entering biological sex and tumor stage as additional fixed factors into a saturated main-effects factorial GLM. Due to the exploratory nature of this study and because the eight SASB clusters represent distinct psychosocial constructs guided by precise prior clinical hypotheses, no formal correction for multiple testing (e.g., Bonferroni) was applied to the bivariate analyses, avoiding an inflation of Type II errors. Consequently, the overall statistical approach of this study remains exploratory. Statistical significance was set at *p* < 0.05 for all analyses. All statistical analyses were conducted using SPSS for Windows, version 24.0 (SPSS Inc., Chicago, IL, USA).

## 3. Results

### 3.1. Socio-Demographic Characteristics

The characteristics of the 2 patient subgroups (adult vs. older) are summarized in Table 1.

The two age groups (adults <65 years and older patients ≥65 years) were comparable in terms of gender distribution (*p* = 0.280) and cancer stage (*p* = 0.138). Regarding educational level, none of the participants were classified as illiterate; therefore, this specific category was not represented in the study sample and was not reported in Table 1. Significant differences emerged in marital status and educational level (both *p* < 0.001), with older patients being more frequently widowed and having lower educational attainment compared to adult patients. These socio-demographic differences suggest that older patients may experience different social and cognitive resources, which could influence intrapsychic and interpersonal functioning.

### 3.2. SASB Intrapsychic Behaviors According to Age

Comparisons between adults (<65 years) and older patients (≥65 years) indicated a significant difference in Cluster 1 (Autonomy—Assertive and separating), with older patients scoring higher (4.4 vs. 3.9; *p* = 0.002). This univariate difference indicates higher scores in autonomy-related items within the older group at the univariable step. No significant differences were observed in the remaining clusters (Clusters 2–8), indicating that other intrapsychic behaviors were largely similar across age groups (Table 2).

When the association between age and Cluster 1 was further examined within a multivariable framework, an adjusted factorial General Linear Model (factorial GLM) was performed, incorporating marital status, educational level, and their respective interaction terms as factors. The comprehensive results of this exploratory post hoc factorial model are detailed in Table 3. When fully accounting for these variables and their interactions, the main effect of the age cohort (<65 vs. ≥65 years) was no longer statistically significant (F = 3.751, *p* = 0.054, partial eta-squared = 0.015). Crucially, neither the main effect of marital status (*p* = 0.086), education (*p* = 0.234), nor any of the interaction terms reached statistical significance. This includes the two-way interaction between age group and educational level, which was verified to be non-significant (F = 1.476, *p* = 0.222, partial eta-squared = 0.018). These findings suggest that the initial unadjusted association between age group and Cluster 1 may be attenuated after adjustment for selected socio-demographic variables. No evidence was found that educational level significantly moderates this association.

Furthermore, to address potential residual confounding based on clinical and theoretical plausibility rather than statistical screening alone, a sensitivity analysis was conducted by additionally adjusting the model for biological sex and tumor stage. Neither biological sex (F = 0.089, *p* = 0.765, partial eta-squared < 0.001) nor tumor stage (F = 1.898, *p* = 0.130, partial eta-squared = 0.022) was significantly associated with Cluster 1 scores. The inclusion of these variables left the age-group effect largely unchanged (F = 2.196, *p* = 0.140, partial eta-squared = 0.008). Overall, the sensitivity analysis yielded comparable results, suggesting that the primary findings were not materially affected by additional adjustment for these clinically relevant variables.

## 4. Discussion

The present study examined age-related differences in intrapsychic personality functioning (“Oneself”) in a sample of cancer patients using the SASB model. In particular, the significant difference observed at the bivariate level concerned Cluster 1 (Autonomy-Assertive and Separating), with higher scores observed in older patients. This between-group difference suggests that older patients in our sample showed higher scores on autonomy-related intrapsychic behaviors at the unadjusted level. Although not directly equivalent to psychological acceptance, SASB Cluster 1 behaviors have been conceptualized as reflecting greater emotional flexibility and adaptive self-regulation, which may support more effective coping processes in clinical populations [24]. This pattern may reflect adaptive self-acceptance and spontaneity, as well as a greater focus on personally relevant goals and priorities, consistent with changes in motivational orientation described across adulthood [11,25,26]. These findings suggest that the observed differences in specific intrapsychic behaviors across age groups may reflect not only chronological age but also the broader socio-demographic and life-course contexts that characterize different generations.

However, a crucial methodological caveat emerged when these socio-demographic variables were simultaneously controlled for in our exploratory post hoc multivariable adjusted factorial General Linear Model (factorial GLM). Upon entering marital status, educational attainment, and their interaction terms into the model, the association between age group and Cluster 1 scores was attenuated, with the main effect of age group no longer reaching statistical significance (*p* = 0.054). This statistical attenuation suggests that the observed age-group difference in intrapsychic autonomy may not be explained by chronological age alone. Rather, the initial unadjusted association between age group and Cluster 1 was attenuated after adjustment for selected socio-demographic characteristics, suggesting that cohort-specific socio-demographic factors may have contributed to the observed difference. In line with this interpretation, the observed differences in marital status and educational level between the two groups provide an important explanatory context. Older patients were more frequently widowed and had lower educational attainment, both of which may closely interact with autonomy-related intrapsychic processes. In particular, widowhood may represent an important contextual factor potentially related to autonomy-related intrapsychic processes, as the loss of a partner may involve significant changes in daily roles, social relationships, and patterns of support [27,28]. Educational level, on the other hand, likely reflects cohort-specific differences in access to education rather than stable psychological characteristics. In earlier generations, limited access to prolonged education and earlier entry into the workforce were more common; thus, educational attainment in this sample primarily reflects historical and social conditions rather than individual psychological traits [29,30]. These life-course and cohort-related factors co-occur with the differences observed in Cluster 1 and may contribute to the expression of autonomy-related behaviors across the lifespan. The multivariable analysis showed that once these social and structural determinants are accounted for, the baseline age-group difference is no longer statistically significant, as the age-by-education interaction did not reach statistical significance (*p* = 0.222). These findings suggest that the initial unadjusted association was attenuated after adjustment for selected socio-demographic variables, highlighting that psychosocial profiles are closely intertwined with cohort-specific historical and social backgrounds rather than being driven by chronological age as an isolated factor.

Accordingly, lower educational attainment should not be interpreted as being directly associated with greater psychological acceptance or autonomy, but rather as reflecting cohort-specific historical and social opportunities, including differences in access to education and occupational pathways. In line with Carstensen’s socioemotional selectivity theory, such differences may be associated with a shift toward a more present-focused orientation and a more selective and present-oriented style of self-regulation [31]. Overall, these results highlight the importance of considering socio-demographic and biographical contexts alongside chronological age in psycho-oncological research, as age operates as a broad indicator of a specific developmental stage characterized by distinct social and cohort profiles [32].

The absence of significant differences across most SASB intrapsychic clusters between adult and older cancer patients may reflect the relatively stable nature of intrapsychic personality functioning in adulthood. Core self-related dimensions such as self-regulation, self-criticism, and self-support tend to remain relatively stable over time and are less influenced by chronological age. In addition, the experience of cancer itself may act as a homogenizing stressor, reducing variability across age groups by imposing shared psychological challenges regardless of developmental stage [33,34]. Socio-demographic and contextual factors, such as those observed in our sample, appear closely intertwined with these intrapsychic patterns in this clinical population.

Personality functioning plays a central role in shaping psychological responses to cancer and its treatment. Our findings suggest that these patterns are embedded within contextual and social determinants that vary between age groups. This is consistent with evidence indicating that aging is not necessarily associated with psychological decline [35]. On the contrary, many older adults maintain high levels of psychological well-being and remain actively engaged in social, familial, and community roles, highlighting the importance of considering functional capacity and social embeddedness in psycho-oncological assessment.

From this perspective, a lifespan approach appears useful for interpreting psychological functioning in cancer patients. By conceptualizing age as a marker of developmental context rather than a rigid categorical boundary, this framework allows a more nuanced understanding of individual differences in adaptation to illness, emphasizing the interaction between developmental stage, life experiences, and social roles.

Finally, some limitations of this study must be acknowledged. First, the cross-sectional design does not allow causal or longitudinal inferences regarding the relationship between age group, socio-demographic factors, and personality functioning.

Second, because the selection of Cluster 1 for multivariable adjustment was data-driven, based on univariable baseline differences rather than an a priori designation of a single primary outcome, this specific analytical phase must be interpreted within its exploratory, post hoc boundaries. Although tumor stage was carefully recorded and statistically evaluated, other potentially relevant clinical variables–such as specific cancer types, active treatments received (e.g., chemotherapy, radiotherapy, immunotherapy), time since diagnosis, presence of recurrence, comorbidities (e.g., patients with psychiatric history), and objective functional status-were not originally available in our database. Consequently, these factors could not be entered as covariates in our adjusted models, and residual confounding cannot be excluded. Furthermore, the study was conducted within a single regional healthcare setting, and the heterogeneous composition of the oncological sample may limit the generalizability of the findings. Psychological functioning was assessed through self-report measures, and no formal cognitive screening was available, which may have influenced the accuracy of self-reported responses, particularly among older participants. Given that clinical trajectories and contextual factors can significantly influence patients’ emotional resources, future longitudinal studies are warranted to systematically track oncological variables, cognitive functioning, and intrapsychic profiles over time.

In conclusion, intrapsychic functioning in cancer patients is closely linked to a complex matrix of socio-demographic and life-course factors, suggesting that chronological age should be interpreted in light of the specific social and historical contexts of the patients.

From a clinical perspective, these findings highlight the importance of considering intrapsychic functioning within the broader psychosocial and life-course context of cancer patients. The identification of age-related patterns that are closely intertwined with socio-demographic and biographical factors may supplement more individualized psychological assessment and support strategies in oncology settings [36]. Our findings suggest the relevance of interventions that are sensitive to patients’ personal histories, relational contexts, and individual resources. Future studies should investigate whether interventions targeting emotional regulation, self-awareness, and intrapsychic behaviors may contribute to improving psychological adjustment to cancer and aging. Future research should further examine how such integrative approaches interact with intrapsychic personality functioning to optimize adjustment to cancer and aging.

## 5. Conclusions

Our findings indicate that age-related differences in the intrapsychic functioning of cancer patients are closely intertwined with cohort-specific socio-demographic and life-course factors. Rather than operating as an isolated chronological variable, age distinguishes specific psychological patterns that co-occur with distinct social and biographical profiles, such as marital history and educational background. These findings are supported by our multivariable analysis, where the standalone effect of age was no longer statistically significant after adjusting for selected socio-demographic variables, and no significant interaction effects emerged. This frames our findings within an exploratory framework of attenuation, cautioning against definitive developmental conclusions. This highlights the need for individualized, context-sensitive approaches in psycho-oncological assessment and care, moving beyond rigid age-based interpretations to embrace a holistic view of the patient’s life course and social background.

## Figures and Tables

**Table 1:** Socio-demographic characteristics of the patients across disease subgroups by age.

Variable	Adult Patients (<65 Years) *n* = 141	Older Patients (≥65 Years) *n* = 132	*p*-Value
Age (years), mean ± sd	51.1 ± 9.4	73.4 ± 6.7	<0.001
Patient’s gender, *n* (%)			0.280
Female	84 (59.6)	87 (65.9)	
Male	57 (40.4)	45 (34.1)	
Tumor stage, *n* (%)			0.138
Stage I	18 (12.8)	9 (6.8)	
Stage II	32 (22.7)	27 (20.5)	
Stage III	44 (31.2)	36 (27.3)	
Stage IV	47 (33.3)	60 (45.5)	
Marital status, *n* (%)			<0.001
Single	14 (9.9)	5 (3.8)	
Married	106 (75.2)	92 (69.7)	
Widowed	5 (3.5)	28 (21.2)	
Divorced/separated	16 (11.3)	7 (5.3)	
Educational level, *n* (%)			<0.001
Elementary	23 (16.3)	68 (51.5)	
Middle School	34 (24.1)	26 (19.7)	
High School	54 (38.3)	31 (23.5)	
University	30 (21.3)	7 (5.3)	

**Table 2:** Comparison between adults (aged 40 to 64 years) and older (≥65 years) patients for SASB clusters.

SASB Clusters	Age				
	<65 Years (*n* = 141) Mean ± SD	≥65 Years (*n* = 132) Mean ± SD	95% CI of the Difference	*p*-Value	Cohen’s d
**1**	3.9 ± 1.6	4.4 ± 1.1	(−0.9; −0.2)	0.002	−0.38
**2**	6.1 ± 1.6	6.2 ± 1.4	(−0.5; 0.2)	0.465	−0.09
**3**	6.1 ± 1.4	5.8 ± 1.3	(0.1; 0.6)	0.052	0.24
**4**	5.8 ± 1.5	5.6 ± 1.5	(−0.1; 0.6)	0.178	0.17
**5**	5.2 ± 1.5	5.1 ± 1.3	(−0.2; 0.4)	0.546	0.08
**6**	1.6 ± 1.8	1.6 ± 1.7	(−0.4; 0.4)	0.964	0.01
**7**	1.6 ± 1.5	1.4 ± 1.4	(−0.2; 0.5)	0.473	0.08
**8**	2.2 ± 1.4	2.1 ± 1.4	(−0.2; 0.5)	0.350	0.11

**Table 3:** Factorial General Linear Model for SASB Cluster 1.

	Sum of Squares	df	Mean Square	F	*p*-Value	Partial Eta-Squared
**Corrected model**	86.5	26	3.3	1.7	0.018	0.155
**Age group**	7.2	1	7.2	3.7	0.054	0.015
**Marital status**	12.8	3	4.3	2.2	0.086	0.026
**Education**	8.3	3	2.8	1.4	0.234	0.017
Age group * marital status	3.7	3	1.2	0.6	0.589	0.008
Age group * education	8.5	3	2.8	1.5	0.222	0.018
**Error**	471.8	246	1.9			

“*” indicates interaction effects between factors in the factorial General Linear Mode.

## Data Availability

The data presented in this study are not publicly available due to ethical and legal considerations related to participant confidentiality. However, de-identified datasets may be made available from the corresponding author and principal investigator upon reasonable request.

## Supplementary Materials

The following supporting information can be downloaded at: https://www.mdpi.com/article/10.3390/healthcare14162452/s1, Description of the 8 clusters of “Oneself”-Intrapsychic experience.
