# Supplementary material for: Does Age Matter? A Comparison of Personality Profiles in Adult and Older Patients with Cancer Using the Structural Analysis of Social Behavior (SASB)
Source: Healthcare (Basel). 2026 Aug 8;14(16):2452. doi: 10.3390/healthcare14162452 (PMC13512621; doi:10.3390/healthcare14162452)
Supplement: Supplementary file 1 [file healthcare-14-02452-s001.zip › healthcare-4393469-supplementary.pdf]

Supplementary Materials

Description of the 8 clusters of "Oneself" - Intrapsychic experience:

SASB-Cl-1 = Autonomy - Assertive and separating. This type is based on what he/she considers necessary at the time. The attitude may be spontaneous with self-acceptance and pleasure in his/her experience or it could be disoriented with little weight given to problems and important choices in life.

SASB-Cl-2 = Autonomy and love - Self-accepting and exploring. This type of person accepts and reacts to his/her deepest feelings, feeling solid, integrated and "together". The desire to be open to feelings generally indicates a state of self-satisfaction and acceptance of weak and strong points.

SASB-Cl-3 = Love - Self-supporting and appreciative. This type is deeply appreciative of him/herself and recognizes the ability to treat, care, console and reconsolidate him/herself. It underlines the capacity for self-esteem and in extreme cases, of self-adoration.

SASB-Cl-4 = Love and control - Self-care and development. This type protects and realistically examines the capacity of being positively self-constructive, actively developing his/her abilities and other important qualities for self-growth. This cluster highlights the use of energy to obtain what is needed and desired.

SASB-Cl-5 = Control - Self-regulating and controlling. This type practises self-control. Great self-control is practised for chosen objectives. This may include paying attention to behaviour in order to ensure conforming to ideals, including great activity programmed in order to reach objectives.

SASB-Cl-6 = Control and hate - Self-critical and oppressive. This type of cluster identifies someone who oppresses him/herself and accuses him/herself of inadequacy, evoking feelings of self-guilt and shame. Feelings of uncertainty and guilt can be used by false induction preventing recognition of what is useful and good for the person. This could be self-punitive behaviour, sometimes sufficiently destructive to require therapeutic intervention.

SASB-Cl-7 = Hate - Self-refusing and annulling. This is a self-destructive type who ignores illnesses and wounds, and self-exhaustion. This cluster is implicative of self-refusal and self-deprivation as well as self-inflicted cruelty. Such self-destructive behavior calls for serious qualified psychotherapeutic intervention.

SASB-Cl-8 = Hate and autonomy - Self-negligent and mentally absent. This cluster identifies someone who may daydream, subsequently not developing his/her abilities and

potentials to their full extent. In extreme cases, these individuals are unreasonable and have unjustified ideas, regarding behaviour without any criterion and falling into self-destructive situations. In these cases, it could be beneficial to examine the danger of self-destructive behaviour with a therapist.
